# Association between Adherence to the Antioxidant-Rich Mediterranean Diet and Sensory Processing Profile in School-Aged Children: The Spanish Cross-Sectional InProS Project

**DOI:** 10.3390/nu11051007

**Published:** 2019-05-02

**Authors:** Eva-María Navarrete-Muñoz, Paula Fernández-Pires, Silvia Navarro-Amat, Miriam Hurtado-Pomares, Paula Peral-Gómez, Iris Juárez-Leal, Cristina Espinosa-Sempere, Alicia Sánchez-Pérez, Desirée Valera-Gran

**Affiliations:** 1 Occupational Therapy Area, Department of Surgery and Pathology, Universidad Miguel Hernandez, 03550 Alicante, Spain; enavarrete@umh.es (E.-M.N.-M.); paula.fernandezp@umh.es (P.F.-P.); snavarro@umh.es (S.N.-A.); mhurtado@umh.es (M.H.-P.); pperal@umh.es (P.P.-G.); ijuarez@umh.es (I.J.-L.); c.espinosa@goumh.umh.es (C.E.-S.); 2 Centro de Investigación Biomédica en Red de Epidemiología y Salud Pública (CIBERESP), 28029 Madrid, Spain; 3 Unit of Nutritional Epidemiology, Department of Public Health, Universidad Miguel Hernandez, 03550 Alicante, Spain; 4 Alicante Institute for Health and Biomedical Research (ISABIAL-FISABIO Foundation), 03550 Alicante, Spain

**Keywords:** Mediterranean diet, sensory processing, short sensory profile, tactile sensitivity, taste/smell sensitivity, low energy/weak

## Abstract

We assessed the association between adherence to the Mediterranean diet (MD) and sensory processing in 583 Spanish children aged 3–7 years from the InProS project in Alicante, Spain. Child sensory processing was measured using the short sensory profile (SSP); atypical sensory performance was defined as SSP total score <155; tactile sensitivity <30; taste/smell sensitivity <15; movement sensitivity <13; under-responsive/seeks sensation <27; auditory filtering <23; low energy/weak <26; and visual/auditory sensitivity <19 scores. Adherence to the MD was measured using the Mediterranean diet quality index KIDMED. Multiple Poisson regression models with robust variance, based on the Huber sandwich estimate, were used to obtain prevalence ratios (PR). Our findings suggested that a lower prevalence of atypical tactile and taste/smell sensitivity were associated with having medium (PR = 0.50, 95% CI: 0.25; 0.99; PR = 0.57, 95% CI: 0.33; 0.99, respectively) and high adherence to the MD (PR = 0.58, 95% CI: 0.34; 0.99; PR = 0.33, 95% CI: 0.19; 0.60, respectively), and of atypical low energy/weak with having medium adherence to the MD (PR = 0.37, 95% CI: 0.16; 0.83). A two-point increase in adherence to the MD showed a general positive effect against atypical sensory performance, although it was statistically significant on taste/smell sensitivity (PR = 0.71, 95% CI: 0.59; 0.85) and low energy/weak (PR = 0.80, 95% CI: 0.64; 0.99) subscales. To our knowledge, this is the first study that shows a protective effect of adherence to the MD against prevalence of atypical sensory processing in school-aged children. Further research from longitudinal studies is required to confirm these findings.

## 1. Introduction

The Mediterranean diet (MD) is a dietary pattern characterized by a high intake of foods and fat with important antioxidant properties, such as fruits and vegetables, olive oil, and nuts [1]. Consistent evidence has reported that a higher adherence to the MD has a beneficial effect on multiple health outcomes in adults [2], such as cardiovascular disease [3,4], cancer [5,6], mortality [7], and cognitive health [8], among others. However, research on the relationship between the adherence to the MD and childhood health outcomes remains scant [9], especially in early childhood. 

Preschool to early school age is a critical window for establishing food preferences and developing sensory food aversion, such as neophobia and picky eating (i.e., acceptance of a limited variety of foods), which can lead to refuse to eat specific foods with particular tastes, textures, smells, or appearances [10]. Existing literature has documented that feeding difficulties are associated with less healthy food choices reducing the consumption of fruits, vegetables, and protein foods [11,12,13,14,15]. An unbalanced diet during childhood can have an important impact on children’s health and development, resulting in detrimental consequences on several metabolic outcomes and a higher risk of developing neurological diseases in later adult life if not treated properly [16,17,18,19]. 

Child feeding difficulties may be linked to sensory processing disturbances. Sensory processing is the ability that our brain has to integrate information received from our sensory organs to produce an appropriate and efficient behavioral response [20]. As such, it involves the external and internal senses. Smell, taste, touch, proprioception, vision, hearing, and sound filtering provide information to the brain that has to process and analyze during the eating experience [21,22]; an over or under response to a particular sensory input may indicate sensory processing difficulties. Several studies conducted in children with autism disorders or chronical disease have suggested that sensory processing difficulties may be related to malnutrition and poor diets [21,23,24,25]. In this sense, apart from the likely grave consequences on a child’s health resulting from an unbalanced diet, it should be noted that sensory processing difficulties may have a negative effect on mood, the ability to perform daily functions, and learning [20]. In specific populations, the prevalence rates may range between 80% and 100%, although current research suggest that about one in six children from the general population may be identified with sensory processing difficulties [20].

To our knowledge, there is no previous evidence about the relationship between sensory processing difficulties and dietary quality as measured by the adherence to the MD in children from the general population. Since sensory interventions can help to avoid sensory food aversion and increase the intake of some foods such as fruit and vegetables in preschool children [26,27,28], the understanding of this relationship may yield helpful information to improve the design of interventions aimed at correcting deviations to prevent unhealthy consequences in child’s health and many long-term health problems in later life. Therefore, the purpose of the present study was to assess the association between the adherence to the MD (as well as its components) and sensory processing in a population-based sample of children aged 3–7 years. 

## 2. Materials and Methods

### 2.1. Design and Procedure

The InProS (Infancia y Procesamiento Sensorial (Childhood and Sensory Processing)) project is a population-based cross-sectional study of children aged 3–7 years recruited from a random selection of 21 schools located in the Alicante province, Spain. Recruitment took place between February and May 2016. After getting the permission from the principal of each school, approximately 1700 eligible children were invited to participate in this study through an invitation letter addressed to their parents that contained a participant information sheet outlining the project details, a booklet with several questionnaires, and the instructions on how to complete them. All children were asked to return the informed written parental consent and the questionnaires filled out. A total sample of 620 children returned the documentation required, rendering a response rate of approximately 37%. After excluding participants with missing data for outcome and exposure variables, a sample consisted of 583 (94%) was finally included in the present analysis. All participants provided informed consent and had no incentive to take part in this study. Ethical approval for this research was obtained from Miguel Hernández University and the research was performed in accordance with the Declaration of Helsinki. 

### 2.2. Study Variables

Child sensory processing was measured by the short sensory profile (SSP), a screening tool used to identify children with sensory processing difficulties. The SSP is a parent report measure that consists of a 38-item questionnaire divided into seven different sections or subscales: tactile sensitivity, taste/smell sensitivity, movement sensitivity, under-responsive/seeks sensation, auditory filtering, low energy/weak, and visual/auditory sensitivity. All items are scored on a one-point to five-point scale (i.e., ranging from 1–always to 5–never). The SSP was based on the sensory profile, the questionnaire created by Dunn (1999) [29], which was cross-culturally adapted and validated in Spanish children [30,31]. We obtained the SSP total score and the score on each subscale by summing up the respective values to classify children’s level of sensory abnormality (typical, probable difference, or definite difference) according to the cut-points proposed by Dunn [29]. Children with an atypical sensory performance were defined as follows: SSP total score <155; tactile sensitivity <30; taste/smell sensitivity <15; movement sensitivity <13; under-responsive/seeks sensation <27; auditory filtering <23; low energy/weak <26; and visual/auditory sensitivity <19 subscales. In our sample, the SSP has a good internal consistency (Cronbach’s α= 0.72–0.76 across scales).

The adherence to the MD was assessed using the Mediterranean diet quality index for children and adolescents (KIDMED) [32]. The KIDMED index is a 16-item questionnaire developed to evaluate dietary habits in children and youths aged 2–24 years. It ranges from 0 to 12 points; the scoring was computed as follows: the items denoting a negative connotation as regards the MD were assigned a value of −1, and a value of +1 was applied to those illustrating a positive aspect. To evaluate the adherence to the MD, the sum of the values of the KIDMED index were divided into tertiles: first tertile (0–7 points), low adherence; second tertile (8 points), medium adherence; third tertile (9–12 points), high adherence. 

In this study, other information about children and parental characteristics was also collected. To the best of our knowledge, there is no evidence on determinants affecting sensory processing in typically developing children, although previous research has suggested that sensory processing impairment is common across children with neurodevelopmental disorders [33,34]. For the present analysis, based on prior knowledge about socio-demographic and lifestyle factors related to child neurodevelopmental outcomes from mother–child longitudinal studies, the following variables were considered as a priori potential confounding factors because of their possible associations with exposure and outcome: maternal and paternal characteristics such as age (in years), country of birth (Spain, other country), level of education (primary or less; secondary; university studies) and employment (yes; no), and child characteristics such as age (in years); sex (female; male); body mass index (calculated as parent-reported weight in kilograms divided by parent-reported height in meters squared), sleep duration (in hours per day), sleep quality (good; poor) as measured by the Spanish version of the pediatric sleep questionnaire [35], TV watching (hours per day), and parent-reported physical activity (not active; moderately active; active/very active).

### 2.3. Statistical Analysis

Descriptive analysis of sociodemographic and lifestyle of parents and their children participants in the study was performed using frequencies and percentages for categorical variables, and median and interquartile range for continuous variables. To compare these characteristics between those children classified as a typical sensory profile (i.e., ≥155 points) and those with an atypical sensory profile (i.e., <155 points), we used Chi-square or Fisher’s exact for categorical variables, and U de Mann Whitney test for continuous variables. 

The association between the adherence to the MD, using total score and score categories (i.e., low, medium and high), and sensory profile (i.e., typical vs atypical performance), using the SSP total score and the score of each subscale, was analyzed by multiple Poisson regression models with robust variance based on the Huber sandwich estimate to obtain prevalence ratios (PR) and their 95% confidence interval (CI) [36,37]. Furthermore, we replicated the same analysis for each component of the KIDMED index. A robust Poisson regression model was used instead of the log-binomial regression model due to it not converging [38]. 

Models were adjusted for potential confounders based on those factors previously identified in literature. Moreover, those variables with *p*-values <0.20 in the bivariate analysis and those whose magnitude of the effect for the exposure of interest changed by >10% following a backward elimination procedure were also included. We did not adjust for paternal variables and child body mass index due to the large number of missing values, although we conducted a further sensitivity analysis to explore the possible influence of these variables as potential confounders. Finally, all models were adjusted for the following child’s characteristics: sex, age, sleep quality, and television watching; and for maternal age, educational level, and country of birth.

To assess the possible effect of dose-response in the categories of the adherence to the MD, linear tests were applied for the KIDMED score as continuous variable (low, medium, and high, coded 1–3).

Several sensitivity analyses were also conducted to evaluate the robustness of the main findings. First, to examine the likely influence of sex and age, we performed stratified analyses by the child’s sex and age group. Moreover, taking into account the potential role of several conditions in sensory performance, we observed the effect of the complete model after excluding those children born preterm (<37 weeks of gestation; *n* = 66), with low birthweight (<2500 g; *n* = 56), and children with some diseases (*n* = 45). The father’s education level and country of origin and the child’s body mass index were also added to the complete model to weigh their possible effect on the findings obtained. Regarding the atypical sensory processing, stratified models by those children classified under probable difference and by those under definite difference for the analyzed SSP subscales were carried out. According to the sensory profile categories defined by Dunn [29], children with probable difference in sensory processing were classified as follows: tactile sensitivity 29–27; taste/smell sensitivity 14–12; and low energy/weak 25–24. Children with a definite difference in sensory processing were considered as follows: tactile sensitivity ≤26; taste/smell sensitivity ≤11; and low energy/weak ≤23. Statistical analyses were conducted with software R, version 3.5.1 (R Core Team. R: A language and environment for statistical computing. R Foundation for Statistical Computing, Vienna, Austria; http://www.r-project.org). The applied statistical tests were bilateral and significance was established at 0.05. 

## 3. Results

### 3.1. Demographic and Lifestyle of Maternal, Parternal, and Child’s Characteristics

In our study, the prevalence of atypical sensory performance in children aged 3–7 was 29.8% (SSP total score <155); 11.5% (tactile sensitivity <30); 15.3% (taste/smell sensitivity <15); 22.8% (movement sensitivity <13); 49.1% (under-responsive/seeks sensation <27); 44.4% (auditory filtering <23); 12.3% (low energy/weak <26); and 26.1% (visual/auditory sensitivity <19). The characteristics of the participants included in the study were described in Table 1. With respect to parental features, the median age was around 40 years (38 for the mothers, and 40 for the fathers). More than 80% of parents were Spanish and slightly higher than a third (40.8% of the mothers and 33.1% of the fathers) had university studies. Almost 90% of the fathers reported to be employed while around 30% of the mothers were unemployed. However, mother’s age and employment, and the country of origin and education level of both parents showed statistically significant differences according to the sensory profile of their children. Compared to children with typical sensory performance, children classified as having atypical sensory processing were more likely to have a younger mother and be unemployed, have parents from a foreign country and with non-university studies. Regarding children’s traits, the median age was 5 years and the distribution of boys (50.6%) and girls (49.4%) was almost equal in proportion. Overall, children slept a median of 10 h/day, 10.3% had poor sleep quality, spent a median of 2 h/day watching TV, and 61.4% were physically active/very active. However, sex, sleep quality, TV watching, and physical activity indicated statistically significant differences between sensory processing groups. Children with atypical sensory performance were more likely to be male, have a poor sleep quality, spend more time watching TV, and be physically active or very active, in comparison to those children presenting typical sensory performance.

### 3.2. Association Between Adherence to Mediterranean Diet and Prevalence of Atypical Sensory Processing

The median adherence to the MD was 8 points, 276 (47.3%), 117 (20.1%), and 190 (32.6%) of children were classified as having low, medium, and high adherence, respectively. Table 2 presents prevalence rates of atypical sensory processing for SSP total and subscale scores, as well as the results of the association between the adherence to the MD and the prevalence of atypical sensory processing after adjusting for potential confounders. In general, children who had low adherence to the MD presented the higher rates of atypical sensory processing for SSP total and subscale scores, although children who had medium or high adherence to the MD displayed very similar prevalence rates. Compared with children who had low adherence to the MD, we observed that children who had a medium or high adherence to the MD presented a statistically significant lower prevalence ratio (PR) of atypical tactile sensitivity (PR = 0.50, 95% CI: 0.25; 0.99; PR = 0.58, 95% CI: 0.34; 0.99, respectively), although the association did not show a linear trend effect (*p*-trend = 0.122). A statistically significant lower PR of atypical taste/smell sensitivity was also observed in children who had medium (PR = 0.57, 95% CI: 0.33; 0.99) or high (PR = 0.33, 95% CI: 0.19; 0.60) adherence to the MD, in comparison with those who had low adherence. This association was verified as a strong dose–response relationship (*p*-trend = 0.372 × 10^−3^). Among the remaining SSP subscales, only children who had medium adherence to the MD were less likely to present low energy/weak difficulties (PR = 0.37; 95% CI: 0.16; 0.83), compared with those having low adherence. Moreover, when we explored the association per two-point increase in KIDMED score, the protective effect against the prevalence of atypical sensory processing remained statistically significant for taste/smell sensitivity (PR = 0.71, 95% CI: 0.59; 0.85) and low energy and weak (PR = 0.80, 95%CI: 0.64; 0.99) subscales. Although the association was not statistically significant, a clear positive tendency was also observed between an increment in the adherence to the MD and the SSP total score (PR = 0.90, 95% CI: 0.80; 1.02) and tactile sensitivity (PR = 0.81, 95% CI: 0.64; 1.02). Nevertheless, a positive effect of a higher adherence to the MD on the rest of the SSP subscales was generally evident, although the statistical significance was not reached.

### 3.3. Association between Components of KIDMED Index and Atypical Sensory Processing

Table 3 shows the findings of the association between each component of adherence to the MD as measured by the KIDMED index and the prevalence of atypical sensory processing in the SSP tactile sensitivity, taste/smell sensitivity, and low energy and weak subscales. The estimates showed that children with atypical tactile sensitivity were less likely to have vegetables regularly once a day (PR = 1.61, 95% CI: 1.03; 2.53), have cereals or grains for breakfast (PR = 1.98, 95% CI: 1.29; 3.05), and use olive oil at home (PR = 2.00, 95% CI: 1.02; 3.93). Moreover, although a statistically significant association was not reached, a negative tendency was also observed in these children with respect to eating more than a fruit every day (PR = 1.54, 95% CI: 0.98; 2.44). Children classified as having atypical taste/smell sensitivity were identified as those who mainly did not take a fruit or fruit juice every day (PR = 2.53, 95% CI: 1.69; 3.80), did not have fresh or cooked vegetables regularly once a day (PR = 1.60, 95% CI: 1.08; 2.36), did not consume fish regularly (at least 2–3 times per week), (PR = 1.68, 95% CI: 1.14; 2.48), did not like pulses or eat them more than once a week (PR = 1.87, 95% CI: 1.28; 2.73), not use olive oil at home (PR = 2.21, 95% CI: 1.31; 3.73), although they did consume nuts regularly, at least 2–3 times per week (PR = 0.64, 95% CI: 0.44; 0.93), compared with those children having typical sensory processing. Moreover, children classified as having atypical sensory processing in the low energy/weak subscale were less likely to have fresh or cooked vegetables regularly once a day (PR = 1.64, 95% CI: 1.09; 2.46) and use olive oil at home (PR = 1.97, 95% CI: 1.06; 3.63), compared with those children who presented a typical sensory performance. 

### 3.4. Sensitivity Analyses

Several sensitivity analyses were carried out in order to appraise the robustness of our findings (Table 4). The estimates for tactile sensitivity did not notably change, excepting when we explored the effect only in girls (PR = 0.51, 95% CI: 0.32; 0.80) and, to a lesser extent, when children with poor sleep quality (PR = 0.74, 95% CI: 0.58; 0.94) were excluded from the analysis. The association observed between a two-point increase in adherence to the MD and lower PR of atypical taste/smell sensitivity remained significant and similar in all sensitivity analyses, except when only children aged 5 (PR = 0.84, 95% CI: 0.62; 1.14) and those aged 6–7 (PR = 0.78, 95% CI: 0.55; 1.11) were included, and when those who had poor sleep quality (PR = 0.98, 95% CI: 0.66; 1.46) were excluded, as well as when we only accounted for those children who were classified as having definite atypical sensory processing (PR = 0.87, 95% CI: 0.70; 1.09). By contrast, we observed that the magnitude of the positive effect was higher and statistically significant when including only children aged 3–4 (PR = 0.54, 95% CI: 0.40; 0.73) and those identified as having probable atypical sensory processing (PR = 0.48, 95% CI: 0.37; 0.61). The analyses for low energy/weak subscale did not indicate any substantial change overall, although the estimates did not reach statistical significance. However, we observed a notable attenuation of the main effect, but not significant, when including only children aged 3–4 (PR =1.02, 95% CI: 0.69; 1.50), as well as those children who were classified as having definite atypical sensory processing (PR = 1.04, 95% CI: 0.74; 1.46). On the contrary, the association observed was positively reinforced when including only children aged 6–7 (PR = 0.63, 95% CI: 0.46; 0.86) and those identified as having probable atypical sensory processing (PR = 0.63, 95% CI: 0.47; 0.83). 

## 4. Discussion

In this study, including Spanish children aged 3–7 years, we observed that around a third of children were identified as having atypical sensory performance according to the SSP total score. Although the prevalence of atypical sensory performance differed between the SSP subscales, it should be noted that at least one in nine children presented some difficulty in sensory processing, affecting almost half of children with respect to certain sensory domains. After adjusting for potential confounding factors, our findings showed that a higher adherence to the MD was significantly associated with a lower prevalence of atypical performance in SSP tactile and taste/smell sensitivity and low energy/weak subscales. A lower intake of vegetables and no use of olive oil at home were associated with sensory difficulties as measured by these three SSP subscales. A lower consumption of cereals or grains for breakfast was also related to atypical tactile sensitivity. A lower intake of fruit or fruit juice, fish, pulses, but regular eating of nuts was associated with having atypical taste/smell sensitivity. To our knowledge, this is the first general population-based study that shows that a greater adherence to the MD may be linked to a lower prevalence of sensory processing difficulties in school-aged children. 

Our preliminary hypothesis was that children with atypical sensory processing would present a lower adherence to the MD, compared to children with typical sensory profile. Indeed, we found that the larger rates of prevalence of atypical sensory performance in the SSP total score and nearly all the SSP subscales were observed in children classified as having low adherence to the MD. Since the quality of children’s diet in developed countries constitutes a serious health concern [39], it is important to identify factors that may have an influence on eating behaviors in early life. Previous research has reported that feeding difficulties are very common problems in childhood and are associated with negative effects on dietary intake and dietary variety [14]. Thus, in order to have a better understanding of these early feeding difficulties, and tailor strategies accordingly, alternative approaches based on sensory evaluation have gained broad acceptance among researchers. The interest in sensory processing problems stems for the fact that the process of eating involves integration of sensory domains, differing between individuals with respect to sensitivity to the different properties of food, such as its taste or texture [40,41]. Currently, there is incipient evidence suggesting a connection between sensory processing difficulties and poor eating behaviors [42]. 

Consistent with our findings, few studies showed that eating less fruits and vegetables was associated with sensitivity in tactile and taste/smell domains [22,40,43], but not in the domain of low energy/weak. Although evidence is still insufficient, this later finding may be of particular interest to open new lines of research. The likely explanations accepted so far about the relationship between sensory processing and problematic eating behaviors have been given in terms of acceptance or rejection of foods. In this sense, sensory processing has been postulated as an important contributor to food acceptance [42] due to sensory properties of foods such as tactile, visual, taste, and olfactory characteristics. As far as we know, two previous studies conducted in Spanish children have reported that food neophobia, i.e., reluctance to eat, or avoidance of new foods, was associated with a lower adherence to the MD [44,45], indicating that these children had a poorer dietary quality and less balanced diets. Thus, in connection with the main hypothesis, it may be assumed that a lower adherence to the MD could be related to a poorer health status. As such, the results observed in the domain of low energy/weak draw the attention to the potential impact of the dietary quality on sensory processing. However, the cross-sectional design of this study does not allow us to establish a cause–effect relationship, although it should be noted that our sample population is representative of the general population and the results obtained may be helpful to generate hypothesis that can be further tested via prospective study designs. 

To date, there has been no research that has explored the association between sensory processing and dietary quality. In the present study, we aimed at determining the dietary quality as measured by the adherence to the MD in children in order to assess its effect on the prevalence of atypical sensory performance. Extensive literature has documented that the MD is a dietary pattern that plays a substantial role in the prevention against a wide range of health outcomes, largely due to its antioxidant and anti-inflammatory capacity derived from a great intake of plant foods, fish, and higher use of olive oil [1]. In this regard, our results also supported that the potential protective effect of the MD on sensory processing problems could mainly be attributed to specific foods such as fruits, vegetables, fish, cereals, pulses, and use of olive oil. Nevertheless, strong evidence has reported that the more beneficial effects of the MD lie in the synergistic combination of dietary components of this pattern considered as a whole, resulting in a balanced ratio of n-6 and n-3 essential fatty acids, high oleic acid content, and great amounts of dietary fiber, antioxidants, and polyphenols [46]. Importantly, this may be of particular interest to define strategies to improve the dietary quality in children, suggesting that nutritional interventions based on a MD pattern may be a more suitable option than interventions focused on the increment of only certain foods. In this study, we observed a positive effect of the global diet as measured by adherence to the MD on child sensory outcomes. An increase in the adherence to the MD was significantly associated with a lower prevalence of sensory difficulties in taste/smell sensitivity and low energy/weak domain. In general, a protection against the prevalence of atypical sensory processing as measured by the SSP total score and some SSP subscale scores was also shown, although the statistical association was not significant because of, to a large extent, the lack of statistical power.

This study has several shortcomings that should be considered for the interpretation of the findings. Since all data were self-reported, there could be some misclassification, although any inaccuracy in reporting should be non-differential. In addition, the questionnaires used to collect information from the study participants were valid and reliable instruments employed in previous research. The cross-sectional analysis of our data prevents us from establishing a causal link between adherence to the MD and sensory processing; however, they do constitute a suitable rationale for replicating in other samples using a prospective study design. Moreover, it should be noted that our sample was collected from the general population and randomly selected in order to preserve the representativeness of data. Nevertheless, the results obtained require corroborating in larger samples.

We are aware that our data are far from being able to provide a possible explanation by which higher adherence to the MD may protect against the prevalence of sensory processing difficulties in school-aged children. We adjusted for a wide range of potential confounding factors, although the effect of unknown factors, residual confounding, or bias due to information not collected cannot be disregarded. Finally, we evaluated the robustness of the findings accounting for specific conditions that could interfere or be related to child sensory processing performing several sensitivity analyses.

## 5. Conclusions

This study shows the potential positive effect of a higher adherence to the MD on sensory performance (i.e., SSP total score and almost all SSP scale scores) in children aged 3–7 years. These findings add to other evidence supporting the possible beneficial effect of the MD on child health outcomes. The positive association between a higher adherence to the MD and sensory performance was observed in SSP tactile and taste/smell sensitivity and low energy/weak subscales. This was more evident in taste/smell sensitivity and low energy/weak subscales when exploring the effect as a linear relationship and was notably stronger in taste/smell sensitivity, unlike the rest of SSP subscales. Compared to children with typical sensory performance, children with difficulties in tactile and taste/smell sensitivity and low energy/weak domain presented lower intakes of Mediterranean foods such as vegetables, fruits, fish, pulses, cereals, and olive oil. Nevertheless, our results should be confirmed in further prospective studies; in the meantime, efforts to improve dietary quality in early childhood should incorporate, in line with the whole diet approach for the MD, intervention strategies aimed at promoting the MD food pattern. 

## Figures and Tables

**Table 1:** Demographic and lifestyle of maternal, paternal, and child characteristics according to sensory profile (typical or atypical) of children aged 3–7 from the InProS Project, Alicante, Spain (*n* = 583).

	Total	Sensory Profile ^3^		*p*-Value ^4^
		Typical: *n*; %	Atypical: *n*; %	
		409; 70.2	174; 29.8	
**Maternal characteristics**				
Age (years), median (IR)	38 (35, 41)	38 (35, 41)	37 (33, 41)	0.169 × 10^−2^
Country of birth (Spain), %	84.7	89.2	74.1	0.349 × 10^−5^
Education (university studies), %	40.8	43.8	33.9	0.054
Employment (yes), %	69.3	73.8	59.2	0.563 × 10^−3^
**Paternal characteristics ^1^**				
Age (years), median (IR)	40 (37, 43)	40 (37, 43)	40 (36, 43)	0.149
Country of birth (Spain), %	83.3	87.6	73.0	0.936 × 10^−4^
Education (university studies), %	33.1	36.8	24.2	0.017
Employment (yes), %	89.5	89.8	88.8	0.743
**Child characteristics**				
Age (years), median (IR)	5 (4, 6)	5 (4, 6)	5 (4, 6)	0.342
Sex (female), %	49.4	54.0	38.5	0.785 × 10^−3^
Body mass index ^2^, median (IR)	16.0 (14.5, 17.4)	15.7 (14.3, 17.4)	16.0 (15.0, 17.4)	0.096
Sleep (h/day), median (IR)	10.0 (9.3, 10.3)	10.0 (9.4, 10.3)	10.0 (9.3,10.4)	0.401
Sleep quality (poor), %	10.3	4.4	24.1	0.115 × 10^−11^
TV watching (h/day), median (IR)	2.0 (1.3, 2.6)	1.9 (1.3, 2.3)	2.2 (1.6, 3.0)	0.291 × 10^−4^
Physical activity (active/very active), %	61.4	58.7	67.8	0.041

IR: Interquartile range. ^1^ Paternal information is available for 523 parents; ^2^ Body mass index is available for 460 children; ^3^ Sensory profile of children was determined by the short sensory profile to classify children’s level of sensory abnormality as typical (≥155 points) and atypical (<155 points); ^4^ From the Chi-square test or Fisher’s exact test (categorical variables) and U de Mann-Whitney (continuous variables).

**Table 2:** Association between adherence to the Mediterranean diet (MD) and prevalence of atypical sensory processing using total and subscales scores of short sensory profile (SSP) in children aged 3–7 years from InProS Project, Alicante, Spain (*n* = 583).

	Adherence to Mediterranean Diet (KIDMED)										
	Low (0–7 Points)	Medium (8 Points)			High (9–12 Points)			*p*-Trend ^1^	Two-Point Increase		
	*n* Cases	*n* Cases	PR ^2^ (95% CI)	*p*-Value	*n* Cases	PR ^2^ (95% CI)	*p*-Value		*n* Cases	PR ^2^ (95% CI)	*p*-Value
SSP total score (atypical; <155 points)	100	27	0.77 (0.54; 1.12)	0.175	47	0.83 (0.62; 1.10)	0.187	0.236	174	0.90 (0.80; 1.02)	0.097
Tactile sensitivity (atypical; <30 points)	44	8	0.50 (0.25; 0.99)	0.049	15	0.58 (0.34; 0.99)	0.049	0.122	67	0.81 (0.64; 1.02)	0.079
Taste/smell sensitivity (atypical; <15 points)	64	13	0.57 (0.33; 0.99)	0.048	12	0.33 (0.19; 0.60)	0.257 × 10^−3^	0.372 × 10^−3^	89	0.71 (0.59; 0.85)	0.201 × 10^−3^
Movement sensitivity (atypical; <13 points)	73	22	0.77 (0.51; 1.17)	0.225	38	0.84 (0.60; 1.18)	0.323	0.359	133	0.91 (0.78; 1.06)	0.221
Underresponsive/seek sensation (atypical; <26 points)	148	56	1.03 (0.83; 1.27)	0.822	82	0.90 (0.75; 1.09)	0.291	0.520	286	0.99 (0.92; 1.08)	0.894
Auditory filtering (atypical; <23 points)	131	56	1.11 (0.89; 1.39)	0.357	72	0.88 (0.71; 1.09)	0.254	0.476	259	1.02 (0.93; 1.11)	0.723
Low energy/weak (atypical; <26 points)	47	6	0.37 (0.16; 0.83)	0.015	19	0.79 (0.48; 1.29)	0.344	0.282	72	0.80 (0.64; 0.99)	0.049
Visual/auditory sensitivity (atypical; <19 points)	75	25	0.88 (0.59; 1.30)	0.513	52	1.16 (0.86; 1.56)	0.330	0.358	152	0.96 (0.83; 1.11)	0.578

^1^ To calculate P-trend, the values 0, 1, and 2 were assigned to low, medium, and high categories of the adherence to the MD in order to enter the variable into the model as a continuous term. ^2^ PR: Prevalence Ratio adjusted for children: sex (female; male), age (in years), sleep quality (good; poor) and TV watching (in hours/day), and for mother’s characteristics: age (in years), educational level (primary or less; secondary; university studies), country of birth (Spain; other country); CI: Confidence interval; SSP: Short sensory profile.

**Table 3:** Association between components of KIDMED index and atypical sensory processing in the SSP taste/smell sensitivity and low energy/weak subscales in children aged 3–7 years from the InProS Project, Alicante, Spain (*n* = 583).

	Atypical Sensory Processing					
Accomplishment vs. No Accomplishment	Tactile Sensitivity		Taste/Smell Sensitivity		Low Energy/Weak	
	(<30 Points)		(<15 Points)		(<26 Points)	
	PR ^1^ (CI 95%)	*p*-Value	PR ^1^ (CI 95%)	*p*-Value	PR ^1^ (CI 95%)	*p*-Value
Takes a fruit or fruit juice every day	1.66 (0.94; 2.94)	0.081	2.53 (1.69; 3.80)	0.712 × 10^−5^	0.55 (0.25; 1.21)	0.134
Has a second fruit every day	1.54 (0.98; 2.44)	0.062	1.42 (0.94; 2.15)	0.094	0.87 (0.57; 1.33)	0.515
Has fresh or cooked vegetables regularly once a day	1.61 (1.03; 2.53)	0.038	1.60 (1.08; 2.36)	0.018	1.64 (1.09; 2.46)	0.018
Has fresh or cooked vegetables more than once a day	1.17 (0.72; 1.89)	0.526	1.49 (0.96; 2.31)	0.072	1.17 (0.74; 1.83)	0.502
Consumes fish regularly (at least 2–3 times per week)	0.94 (0.56; 1.60)	0.829	1.68 (1.14; 2.48)	0.009	1.19 (0.77; 1.85)	0.430
Goes more than once a week to a fast-food restaurant ^2^	0.72 (0.40; 1.28)	0.259	0.95 (0.54; 1.69)	0.869	0.71 (0.41; 1.23)	0.224
Likes pulses and eats them more than once a week	1.05 (0.63; 1.74)	0.853	1.87 (1.28; 2.73)	0.001	1.17 (0.75; 1.84)	0.488
Consumes pasta or rice almost every day (≥5 times per week)	0.91 (0.57; 1.44)	0.682	1.03 (0.70; 1.53)	0.868	1.20 (0.77; 1.85)	0.421
Has cereals or grains (bread, etc.) for breakfast	1.98 (1.29; 3.05)	0.002	1.37 (0.93; 2.02)	0.110	1.19 (0.74; 1.90)	0.470
Consumes nuts regularly (at least 2–3 times per week)	0.95 (0.60; 1.51)	0.830	0.64 (0.44; 0.93)	0.020	1.13 (0.74; 1.74)	0.565
Uses olive oil at home	2.00 (1.02; 3.93)	0.043	2.21 (1.31; 3.73)	0.003	1.97 (1.06; 3.63)	0.031
Skips breakfast ^2^	0.69 (0.30; 1.60)	0.391	0.69 (0.35; 1.37)	0.285	0.92 (0.39; 2.18)	0.844
Has a dairy product for breakfast (yoghurt, milk, etc.)	0.70 (0.27; 1.85)	0.478	0.40 (0.13; 1.26)	0.117	0.49 (0.19; 1.28)	0.144
Has commercially baked goods or pastries for breakfast ^2^	1.73 (0.86; 3.49)	0.125	1.24 (0.71; 2.17)	0.449	0.59 (0.34; 1.02)	0.058
Takes two yoghurts and/or some cheese (40 g) daily	0.81 (0.51; 1.31)	0.390	1.08 (0.73; 1.61)	0.687	1.38 (0.90; 2.11)	0.139
Takes sweets and candy several times every day ^2^	1.15 (0.65; 2.06)	0.628	0.82 (0.50; 1.34)	0.430	1.01 (0.63; 1.64)	0.954

^1^ PR: Prevalence ratios were adjusted for child’s characteristics: sex (female; male), age (in years), sleep quality (good; poor) and TV watching (in hours/day); and for mother’s characteristics: age (in years), educational level (primary or less; secondary; university studies), country of birth (Spain; other country); CI: Confidence Interval; ^2^ ”no accomplishment” was the reference category used for these components of the KIDMED index due to these items that were negatively scored with respect to the Mediterranean diet.

**Table 4:** Sensitivity analyses of the multiple-adjusted prevalence ratios for the association between two-point increase of the adherence to the MD and atypical sensory processing in the SSP tactile sensitivity, taste/smell sensitivity, and low energy and weak subscales in children aged 3–7 from InProS Project, Alicante, Spain.

		Atypical Sensory Processing								
	*n*	Tactile Sensitivity			Taste/Smell Sensitivity			Low Energy/Weak		
		(<30 Points)			(<15 Points)			(<26 Points)		
		*n*	PR ^1^ (CI 95%)	*p*-Value	*n*	PR ^1^ (CI 95%)	*p*-Value	*n*	PR ^1^ (CI 95%)	*p*-Value
Complete model	583	67	0.81 (0.64; 1.02)	0.079	89	0.71 (0.59; 0.85)	0.201 × 10^−3^	72	0.80 (0.64; 0.99)	0.049
Including only boys	295	43	1.07 (0.81; 1.41)	0.620	48	0.71 (0.56; 0.90)	0.004	35	0.85 (0.66; 1.09)	0.209
Including only girls	288	24	0.51 (0.32; 0.80)	0.003	41	0.74 (0.55; 1.00)	0.048	37	0.75 (0.53; 1.06)	0.103
Including only children aged 3–4	182	27	0.73 (0.48; 1.11)	0.142	32	0.54 (0.40; 0.73)	0.734 × 10^−4^	24	1.02 (0.69; 1.50)	0.936
Including only children aged 5	196	22	0.81 (0.59; 1.11)	0.183	29	0.84 (0.62; 1.14)	0.267	21	0.84 (0.54; 1.29)	0.428
Including only children aged 6–7	205	18	0.78 (0.46; 1.33)	0.366	28	0.78 (0.55; 1.11)	0.164	27	0.63 (0.46; 0.86)	0.004
Excluding poor sleep quality	461	55	0.74 (0.58; 0.94)	0.014	69	0.68 (0.56; 0.82)	0.838 × 10^−4^	55	0.78 (0.62; 0.99)	0.042
Excluding preterm children	505	53	0.83 (0.64; 1.09)	0.185	75	0.77 (0.63; 0.93)	0.006	56	0.83 (0.64; 1.08)	0.169
Excluding low weight at birth	537	59	0.80 (0.62; 1.02)	0.067	77	0.71 (0.59; 0.86)	0.483 × 10^−3^	62	0.84 (0.66; 1.06)	0.132
Excluding children with some diseases	523	62	0.78 (0.61; 0.99)	0.049	76	0.72 (0.58; 0.88)	0.002	66	0.83 (0.67; 1.03)	0.097
Adjusted for child body mass index	460	53	0.83 (0.65; 1.06)	0.143	67	0.72 (0.60; 0.87)	0.554 × 10^−3^	58	0.83 (0.66; 1.05)	0.118
Adjusted for father’s education and country of birth	523	57	0.87 (0.68; 1.12)	0.274	81	0.70 (0.58; 0.85)	0.394 × 10^−3^	64	0.79 (0.61; 1.02)	0.073
Including only probable atypical sensory processing	531	34	0.83 (0.60; 1.15)	0.261	52	0.48 (0.37; 0.61)	0.619 × 10^−8^	28	0.63 (0.47; 0.83)	0.001
Including only definitive atypical sensory processing	546	33	0.79 (0.58; 1.08)	0.145	37	0.87 (0.70; 1.09)	0.236	44	1.04 (0.74; 1.46)	0.809

^1^ PR: Prevalence ratios were adjusted for child’s characteristics: sex (female; male), age (in years), sleep quality (good; poor), and TV watching (in hours/day); and for mother’s characteristics: age (in years), educational level (primary or less; secondary; university studies), country of birth (Spain; other country); CI: Confidence Interval.
